# FINDABILITY OF HEALTH EQUITY DEFINITIONS ON REPUTABLE AGING AND PUBLIC HEALTH WEBSITES

**DOI:** 10.1093/geroni/igad104.3358

**Published:** 2023-12-21

**Authors:** Mahederemariam Dagne, Elizabeth Terhune, Patricia Heyn

**Affiliations:** Marymount University, Arlington, Virginia, United States; Marymount University, Arlington, Virginia, United States; Marymount University, Arlington, Virginia, United States

## Abstract

In the current era of electronic health information, ensuring proper access to consistent and accurate information supporting health equity is crucial for older adults. It is imperative that older adults comprehend health terminologies and definitions on websites, enabling them to understand the necessary information effectively. To evaluate how public health websites define, communicate, and display health equity terms and definitions, we developed a tool to review how easy it is to find and understand information on websites that play an integral role in guiding the health of older adults. Thus, we reviewed over 60 credible websites widely used for health information. Many of them included dedicated aging content. Our evaluation revealed that health equity definitions were inconsistent across websites and were often challenging to locate, requiring substantial time and effort to retrieve the information. Additionally, 50% of them lacked definitions. Furthermore, most websites failed to include citations that could substantiate the provided definitions. This study underscores the absence of a systematic, standardized, and regulated approach to how public health websites inform the public, particularly by using consistent terms to define health equity. This study highlights the lack of a universally shared and standardized health equity definition for websites. We recommend that websites structure their content to be easily findable and propose adopting universal standards for displaying health information on websites, especially when communicating with the public. This can mitigate confusion, enhance the understanding of health equity, and ensure important health information findability for older adults.

